# Associative Imagery as a Strategy to Improve Destination Memory in Younger and Older Adults

**DOI:** 10.1093/geroni/igaa057.1164

**Published:** 2020-12-16

**Authors:** Tara Johnson, Katie Stanko, Susan Jefferson

**Affiliations:** 1 Indiana University of Pennsylvania, Indiana, Pennsylvania, United States; 2 University of Pittsburgh, Pittsburgh, Pennsylvania, United States; 3 Pittsburgh VA Health Care System, Pittsburgh, Pennsylvania, United States

## Abstract

Destination memory errors (inability to remember to whom information was shared) affects all ages, but older adults are particularly vulnerable due to poor source monitoring. Individuals may assume information was already shared when it was not or repeat previously shared information. The current study explored two mental imagery strategies (vivid imagery, visualizing context) to improve destination memory. Using a software program, younger and older adults told randomly generated facts to random celebrity faces. Participants were unaware of the upcoming memory tests. The control group did not use a strategy. The imagery group used vivid imagery to connect the fact and face (e.g., visualize Oprah on a dime to remember Oprah was told that dimes have 118 ridges). The context group visualized a provided context (e.g., grocery store) when telling a fact to a face. Assessments of performance on item memory (facts, faces) as well as destination memory (face-fact pairings) were counterbalanced. Results indicated an associative memory deficit among older adults, which was driven by a higher rate of false alarms. However, across all adults, the vivid imagery condition was more accurate than the control condition, and they demonstrated fewer false alarms. These findings suggest that older adults can use mental imagery to reduce false alarms and improve destination memory performance. Implications include reducing age stereotypes, improving conversations, and decreasing potentially dangerous situations (e.g., withholding important health information thinking it already was shared with a doctor).

